# RecOR complex including RecR N-N dimer and RecO monomer displays a high affinity for ssDNA

**DOI:** 10.1093/nar/gks889

**Published:** 2012-09-27

**Authors:** Qun Tang, Pu Gao, Yan-Ping Liu, Ang Gao, Xiao-Min An, Shun Liu, Xiao-Xue Yan, Dong-Cai Liang

**Affiliations:** ^1^National Laboratory of Biomacromolecules, Institute of Biophysics, Chinese Academy of Sciences, Beijing 100101 and ^2^Graduate University of Chinese Academy of Sciences, Beijing 100039, China

## Abstract

RecR is an important recombination mediator protein in the RecFOR pathway. RecR together with RecO and RecF facilitates RecA nucleoprotein filament formation and homologous pairing. Structural and biochemical studies of *Thermoanaerobacter tengcongensis* RecR (TTERecR) and its series mutants revealed that TTERecR uses the N-N dimer as a basic functional unit to interact with TTERecO monomer. Two TTERecR N-N dimers form a ring-shaped tetramer via an interaction between their C-terminal regions. The tetramer is a result of crystallization only. Hydrophobic interactions between the entire helix-hairpin-helix domains within the N-terminal regions of two TTERecR monomers are necessary for formation of a RecR functional N-N dimer. The TTERecR N-N dimer conformation also affects formation of a hydrophobic patch, which creates a binding site for TTERecO in the TTERecR Toprim domain. In addition, we demonstrate that TTERecR does not bind single-stranded DNA (ssDNA) and binds double-stranded DNA very weakly, whereas TTERecOR complex can stably bind DNA, with a higher affinity for ssDNA than double-stranded DNA. Based on these results, we propose an interaction model for the RecOR:ssDNA complex.

## INTRODUCTION

The repair of damaged DNA is required to maintain the genomic integrity of living organisms, and the non-homologous end-joining and recombination DNA repair mechanisms occur in all organisms (1,2). Two recombination DNA repair pathways exist in bacteria: the RecBCD pathway and the RecFOR pathway (3–5). The RecBCD pathway is responsible for repairing double-stranded DNA (dsDNA) breaks, whereas the RecFOR pathway is mainly used for single-stranded DNA (ssDNA) gap repair (6–8); Recently, it was shown that the RecF pathway, which has many parallels with recombinational repair in eukaryotes, is important for recombinational repair of DNA breaks and gaps using a reconstituted system (9), and some work suggests that the functions of the proteins involved in the RecFOR pathway are conserved from bacteria to humans (10–14). RecR is the most conserved protein in the RecFOR pathway; RecR binds to RecO, and RecO interacts with SSBs (15–17), then the complex facilitates RecA filament formation on ssDNA-binding protein (SSB)-coated ssDNA (5,16,18–21). When associated with RecF, the RecOR complex is implicated in the recognition of dsDNA–ssDNA junctions (9,22,23).

RecR is a zinc metalloprotein (24), which consists of a helix-hairpin-helix (HhH) motif, zinc finger motif, Toprim domain and C-terminal hydrophobic region. At present, the HhH motif is thought to be required for binding DNA (21,25,26), the zinc finger motif plays a structural or DNA-binding role (21,24) and the RecR Toprim domain is the binding site for both RecO and RecF (23). RecR proteins from different species have different polymerization states in solution (18,21,23,27) and varying abilities to bind DNA (16,21,27,28). The crystal structure of *Deinococcus radiodurans* RecR (drRecR) indicated that a ring-shaped tetramer is the functional unit for DNA binding (21). From the NMR model of *Thermus thermophilus* RecR (ttRecR), Honda *et al.* (23) suggested that ttRecR forms a dimer in solution by N-terminal interactions. Based on the ring-shaped tetramer structure of drRecR and the drRecR: drRecO molecular ratio of 4:2 in solution, Timmins *et al.* (29) obtained a structural model of the drRecOR complex at a resolution of 3.8 Å and speculated a model of RecOR complex with dsDNA. Small-angle X-ray-scattering data have indicated that ttRecFR forms a globular structure consisting of four ttRecR and two ttRecF monomers, and the modularized interaction mechanisms have been speculated for the RecOR complex and RecFR complex with DNA (23,30).

The RecR, RecF and RecO proteins have been intensively researched. The individual crystal structures of RecR, RecO and RecF have been solved, and the regions that are possibly involved in protein–protein and protein–DNA interactions have been identified (17,21,31–34). However, in contrast to the RecBCD pathway, the mechanism of recombination repair mediated by the RecFOR pathway is poorly understood. Establishing the assembly pattern of RecR, RecO and RecF with DNA will promote further study of the RecFOR pathway. In this study, we investigated the structures and biological function of *Thermoanaerobacter tengcongensis* RecR (TTERecR) and its series mutants. Based on these experiments, we proposed a novel interaction model for RecOR:ssDNA complex, which would provide further understanding of the mechanism of RecFOR repair pathway.

## MATERIALS AND METHODS

### Protein purification

The *recR* (TTE0041; GenBank: AAM23354.1), *recF* (TTE0004; GenBank: AAM23321.1) and *recO* (TTE0976; GenBank: AAM24231.1) genes were amplified from *T. tengcongensis MB4* genomic DNA by PCR and individually cloned into the pETDuet plasmid (Novagen) for expression with an N-terminal hexahistidine tag. TTERecR site-specific mutants and deletion mutants were generated from the TTE-recR-pETDuet plasmid. All sequences were confirmed by sequencing. The proteins were over-expressed in *Escherichia coli* BL21 (DE3). The cells were cultured in LB media containing 100 mg/l ampicillin at 37°C for 8 h and induced with 0.4 mM isopropyl β-d-thiogalactoside for 10 h at 28°C. The recombinant proteins were purified by sonication and two-step column chromatography using a Ni-affinity column and Superdex200 gel-filtration column (GE Healthcare). RecR and the RecR mutants were concentrated to 20 mg/ml by ultrafiltration in 10 mM Tris, pH 7.5, 200 mM NaCl, and RecO was concentrated to 20 mg/ml by ultrafiltration in 10 mM Tris, pH 7.5, 1 M NaCl. All proteins were stored at −80°C.

### Crystallization and structure determination

The crystals of TTERecR and its mutants were obtained at 20°C for a few days by the hanging drop vapor diffusion technique. TTERecR was crystallized in buffer containing 6% (w/v) PEG3350, 200 mM lithium citric tribasic tetrahydrate, 100 mM MES, pH 6.8. The TTERecR_16-196 _deletion mutant was crystallized in 14% (w/v) PEG3350, 150 mM ammonium sulfate, 100 mM MES, pH 5.15. The site-specific mutant TTERecR_K21G_ was crystallized in 8% (w/v) PEG4000, 200 mM ammonium sulfate, 10% (v/v) 2-propanol, 100 mM HEPES sodium salt, pH 7.5. The TTERecR_1-166_ deletion mutant was crystallized in 2.1 M sodium formate, 100 mM Bis-Tris propane, pH 7.0. The TTERecR_1-180_ deletion mutant was crystallized in 10% (w/v) PEG6000, 2.0 M NaCl, pH 5.6. The crystals were flash frozen by immersion in a reservoir of 15–25% glycerol followed by transferring to liquid nitrogen. The crystals were maintained at 100 K during X-ray diffraction data collection using the beamline BL17A (TTERecR, λ = 0.9875 Å) or beamline NW12 (TTERecR_16-196_, λ = 0.9875 Å) at the Photon Factory (Tsukuba, Japan), beamline BL17U (TTERecR_K21G_, λ = 1.005 Å) at Shanghai Synchrotron Radiation Facility (Shanghai, China) or beamline 1W2B (TTERecR_1-180_ and TTERecR_1-166_, λ = 1.005 Å) at Beijing Synchrotron Radiation Facility (Beijing, China). The diffraction images were indexed and integrated using HKL2000 (35). The data collection statistics are presented in Table 1.

**Table 1.** Data collection and refinement statistics for TTERecR and its series mutant

	TTERecR_full-length_	TTERecR_K21G_	TTERecR_16-196_
Data collection			
Space group	P21	F222	P21212
Cell dimensions			
a, b, c (Å)	90.3, 68.3, 94.1	66.8, 123.7,135.2	84.4, 84.7, 72.6
α, β, γ (°)	β* = *92.95		
Resolution (Å)	50–2.45 (2.54–2.45)	50–2.9 (3.2–2.8)	50–2.8 (3.0–2.8)
*R*_merge_	6.2 (38.3)	9.7 (46.6)	13.5 (54.3)
*I*/σ*I*	36.7 (4.8)	15.1 (4.2)	39.4 (8.45)
Completeness (%)	97.56 (86.00)	98.21 (100.00)	98.00 (94.00)
Redundancy	7.4 (6.3)	5.8 (6.2)	13.1 (13.3)
Refinement			
Resolution (Å)	20–2.45	20–2.8	20–2.8
No. reflections	41 210	6949	13 020
*R*_work_/*R*_free_	19.6, 22.6	22.7, 26.0	20.1, 25.4
No. atoms			
Protein	6125	1488	2655
Zn	4	1	2
*B*-factors			
Protein	54.65	95.84	56.49
Zn	38.2	82.82	48.84
R.m.s. deviations			
Bond lengths (Å)	0.013	0.012	0.015
Bond angles (°)	1.56	1.49	1.43

### Structure determination and refinement

The structure of full-length TTERecR was solved by the molecular replacement method using PHASER (36) with one monomer of drRecR [Protein Data Bank (PDB):1VDD] as the search model at 20-3 Å resolution. The model was completed and refined using the REFMAC5 refinement (37,38) and COOT (39) at 20-2.45 Å resolution. The structures of the mutants TTERecR_16-196_ and TTERecR_K21G_ were solved using the model of TTERecR, and refined using the REFMAC5 or PHENIX refinement programs (40). All structural images were drawn using PyMOL (http://www.pymol.org/). Detailed crystallographic statistics are shown in Table 1. Coordinates have been deposited into PDB under the accession codes: 3VDP, 3VE5 and 3VDU.

### Ultracentrifugation analysis

Sedimentation equilibrium experiments were performed using a Beckman Optima XL-A analytical ultracentrifuge (Beckman Instruments, Palo Alto, CA) equipped with absorbance optics and an An60 Ti rotor at 20°C for 6 h at 60 000 rpm for TTERecR and TTERecO and 55 000 rpm for the TTERecOR complex. The proteins were concentrated to 1 mg/ml in 5 mM Tris–HCl, pH 7.5, 50 mM NaCl. Data analysis was conducted using the c(*s*) or ls-g*(*s*) method using Peter Shuck’s software program SEDFIT (41).

### Size-exclusion chromatography

Size-exclusion chromatography was performed using a fast protein liquid chromatography system (GE Healthcare) on a Superdex-200 HR 10/300 column at a flow rate of 0.5 ml/min. Full-length TTERecR, TTERecR mutants, TTERecO and the TTERecOR complexes (at concentration of 200 µg/ml) were loaded onto the column equilibrated with 50 mM Tris–HCl, pH 7.5, 300 mM NaCl and eluted using the same buffer. Protein elution was monitored by measuring the absorbance at 280 nm. Data analysis was conducted using UNICORN version 5.11 software program.

### Surface plasmon resonance assays of protein with protein

Surface plasmon resonance (SPR) experiments were performed using a Biacore3000 machine (GE Healthcare) at 25°C. One flow cell of the CM5 sensor chip was activated with a 1:1 mixture of 0.2 M EDC and 0.05 M NHS in water, as described by the manufacturer, then 10 ug/ml TTERecO was injected over the flow cell in 10 mM sodium acetate (pH 5.5) at a flow rate of 10 µl/min. The remaining binding sites were blocked using 1 M ethanolamine, pH 8.5, and then 4900 response units (RU) of TTERecO were immobilized. TTERecR and the mutants (ranging in concentration from 0.0125 µM to 4 µM) were injected for 2 min at a flow rate of 30 µl/min. The running buffer was the same as the protein buffer (20 mM HEPES, pH 7.4, 200 mM NaCl and 0.005% [v/v] Tween 20); 20 mM NaOH was injected for 1 min to regenerate the chip surface. Non-specific binding to a blank flow cell was subtracted to obtain corrected sensorgrams; all data were analysed using BIAevaluation software version 4.1 and fitted to a 1:1 (Langmuir) binding model (steady state affinity model) to obtain equilibrium constants.

### SPR assays of protein with DNA

Binding of protein to DNA was investigated using the BIAcore 3000 SPR machine (GE Healthcare) at 25°C. The running buffer (20 mM HEPES, pH 7.4, 200 mM NaCl and 0.005% [v/v] Tween 20) was prepared, vacuum filtered and degassed immediately before use. Biotin-labeled 60 mer ssDNA (5′-[Bio] ATAAATATCgATgTTAAAgAggATAAgATTTATAAAATAgCTTCTTATTTTCCTgTAgTA-3′) and biotin-labeled 60 mer dsDNA (biotin-labeled 60 mer ssDNA mixed with the corresponding 5′-TACTACAggAAAATAAgAAgCTATTTTATAAATCTTATCCTCTTTAACATCgATATTTAT-3′ oligonucleotide) were anchored to the surface of the SA chip (55 RU ssDNA and 89 RU dsDNA). Different concentrations (0.03–9 µM) of the proteins and mutants were injected onto the DNA surface or a blank flow cell for 3 min at a flow rate of 40 µl/min. After a 3–4 min dissociation phase, the remaining proteins were removed using 30 µl 0.03% SDS (v/v). Non-specific binding to the blank flow cell was subtracted to obtain corrected sensorgrams. Equilibrium and kinetic constants were calculated by a global fit to 1:1 (Langmuir) binding model using BIAevalution software version 4.1.

### Electrophoretic gel mobility shift assays

A total of 2 µM substrate DNA (60 mer ssDNA, 60 mer dsDNA or plasmid DNA) was incubated with full-length TTERecR, TTERecR mutants, TTERecO or TTERecOR complex (1, 2, 4 and 10 µM) in 10 µl reaction buffer (20 mM HEPES, pH 7.4, 200 mM NaCl) at 25°C for 30 min. The ssDNA and dsDNA sequences were the same as those used for SPR. Proteins incubated with the 60 mer DNA complex were loaded onto 6% non-denaturing polyacrylamide gels with TBE buffer, and proteins incubated with the plasmid DNA complex were loaded onto 1% agarose gels with TBE buffer. The DNA and DNA–protein complexes were visualized using a SYBR Green EMSA (Molecular Probes EMSA Kit, Invitrogen).

## RESULTS

### TTERecR is a dimer in solution

**Figure 1.**
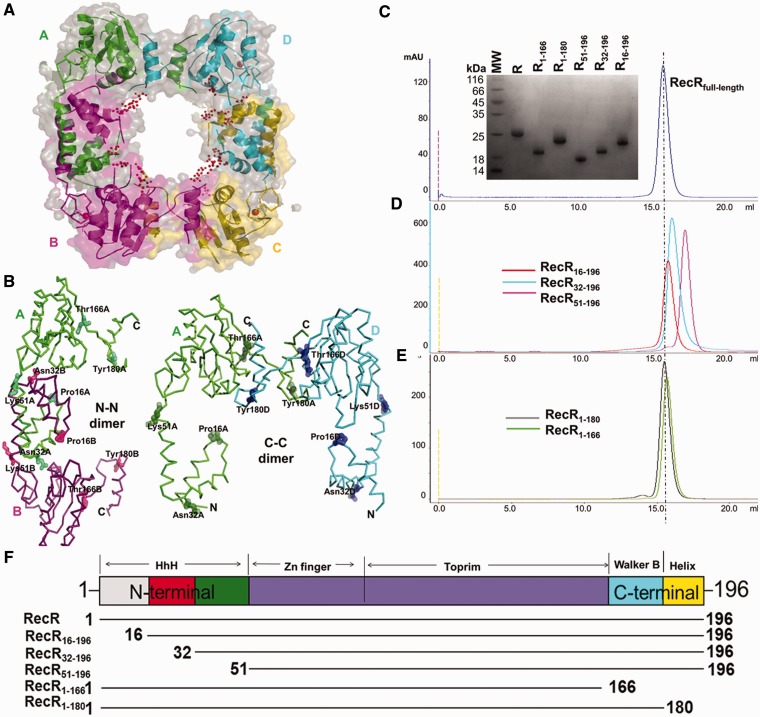
Dimerization interface analysis of TTERecR. (**A**) Sphere model structure of the TTERecR tetramer and the putative DNA-binding region of the central hole. (**B**) Ribbon model structure of the TTERecR A-B monomer dimer (N-terminal interface) and A-D monomer dimer (C-terminal interface). (**C–E**) Size-exclusion chromatography analysis of full-length TTERecR (C), the N-terminal deletion mutants TTERecR_16-196_, TTERecR_32-196_ and TTERecR_51-196 _(D) and the C-terminal deletion mutants TTERecR_1-180_ and TTERecR_1-166 _(E). Size exclusion chromatography was performed using a HiLord 16/60 Superdex 200 column (GE Health Life Sciences) at 0.5 ml/min in 50 mM Tris-HCl, pH 7.5, 300 mM NaCl. Protein elution was monitored by measuring absorbance at 280 nm. The peak fractions of each sample were analysed on Coomassie Blue R-250-stained reducing 15% SDS-polyacrylamide (w/v) gels (inset) and compared with Low MW Protein Ladder (MW; Biomed). (**F**) Schematic domain organization of full-length TTERecR and the TTERecR mutants. The amino acid sequence boundaries of the TTERecR constructs are indicated below.

The crystal structure of TTERecR was solved at a resolution of 2.45 Å by molecular replacement using the model of drRecR (PDB code: 1VDD). The structures of TTERecR and drRecR exhibited similar folds. Four TTERecR monomers (A/B/C/D) in the asymmetric unit formed a ring-shaped tetramer with a central hole of 30–36 Å diameter (Figure 1A). Two types of intersubunit interactions were observed between the neighboring TTERecR: the N-N dimer (A-B; C-D) and the C-C dimer (A-D; B-C) (Figure 1B).

However, size-exclusion chromatography and ultracentrifugation analysis proved that TTERecR [molecular weight (MW): 23.5 kDa] forms a stable dimer (47 kDa) in solution (Figure 1C; Supplementary Figure S2A). Investigation of the RecR dimerization interface pattern is crucially important to study the biological function of this protein (21,27,29,30,42). We constructed a series of N-terminal and C-terminal deletion mutants to investigate the dimerization interface of TTERecR (Figure 1B and F). The TTERecR_1-166 _(MW: 18 kDa) and TTERecR_1-180 _(MW: 20 kDa) C-terminal deletion mutants also formed stable dimers in solution, with molecular masses of 36 kDa and 40 kDa, respectively (Figure 1E). We obtained crystals of the TTERecR_1-166 _and TTERecR_1-180 _mutants (Supplementary Figure S1A and B). Unfortunately, it was difficult to solve these crystal structures because of dozens of TTERecR mutants in an asymmetric unit (Supplementary Figure S1C and D; Supplementary Table S1). Size-exclusion chromatography analysis of the N-terminal deletion mutants showed that TTERecR_16-196 _(MW: 20 kDa) formed a 40 kDa dimer in solution, whereas both TTERecR_32-196_ (MW: 19 kDa) and TTERecR_51-196_ (MW: 16 kDa) were monomeric (Figure 1D). These results indicate that TTERecR cannot form a C-C dimer, when the N-terminus has been truncated in TTERecR_32-196 _and TTERecR_51-196_, even though the C-terminus is still intact. Furthermore, the crystal structure of TTERecR_16-196 _revealed that TTERecR_16-196 _formed an N-N dimer, not a C-C terminal interaction (Supplementary Figure S3A and B). Therefore, the biochemical and structural biology analysis demonstrated that the N-terminus is responsible for dimerization of TTERecR.

### Molecular ratio of TTERecR interacting with TTERecO is 2R:1O

Recent reports indicated that the ring-shaped tetramer of RecR was the basic unit for interaction with two RecO monomers or two RecF monomers (23,29,30). We used size-exclusion chromatography and ultracentrifugation analysis to investigate formation of the TTERecR and TTERecO complex. TTERecO formed a monomer in solution, TTERecR formed a dimer in solution, and the calculated molecular mass of the TTERecOR complex was 78 kDa, corresponding to 2RecR:1RecO (Figure 3G; Supplementary Figure S2B and C).

**Figure 2.**
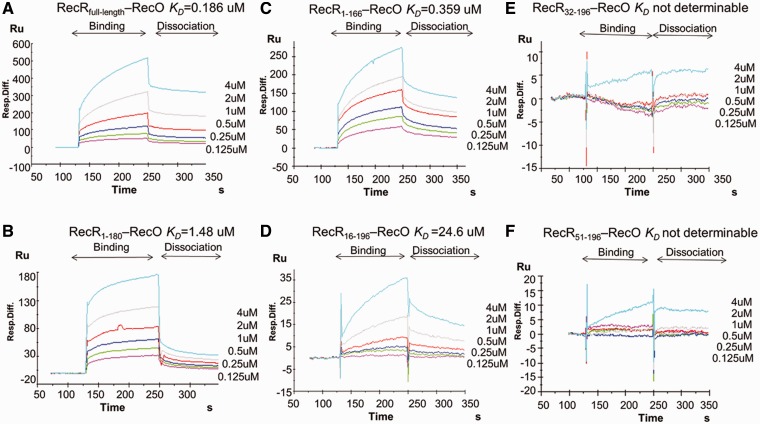
Contribution of the TTERecR N-N dimer to TTERecO binding. BIAcore biosensor analysis of full-length TTERecR (**A**); TTERecR_1-180_ (**B**); TTERecR_1-166 _(**C**); TTERecR_16-196 _(**D**); TTERecR_32-196_ (**E**); TTERecR_51-196_ (**F**), binding to TTERecO at 25°C. Sensorgrams are shown for 0.125, 0.25, 0.5, 1, 2 and 4 µM TTERecR or TTERecR mutants injected over the TTERecO-coupled surface. The apparent *K*_D_ values were calculated from the kinetic *K*_D_ (M) = *K*_d_/*K*_a_.

To further analyse the interaction between the TTERecR dimer and TTERecO monomer, we used the SPR assay to investigate the interactions between TTERecO and the N-terminal and C-terminal deletion mutants of TTERecR (Figure 1F). The binding affinities (*K*_D_) of TTERecO for TTERecR_1-166_ and TTERecR_1-180_ were 0.359 µM and 1.48 µM, respectively; these values were not significantly different to that of binding affinity of full-length TTERecR for TTERecO (*K*_D_ = 0.186 µM; Figure 2A–C). These data indicate that the C-terminus hydrophobic helix region, which forms the C-terminal interaction, does not contribute to the binding of TTERecR with TTERecO. Size-exclusion chromatography assay indicated that TTERecR_32-196 _and TTERecR_51-196 _exist as a monomer in solution with no binding affinity for TTERecO, owing to the loss of the N-N terminal interaction (Figure 1D; Figure 2E and F). These results demonstrate that the N-N dimer of TTERecR is the basic functional unit for interacting with a single TTERecO.

### The RecR N-N dimer is crucial for the interaction with RecO

**Figure 3.**
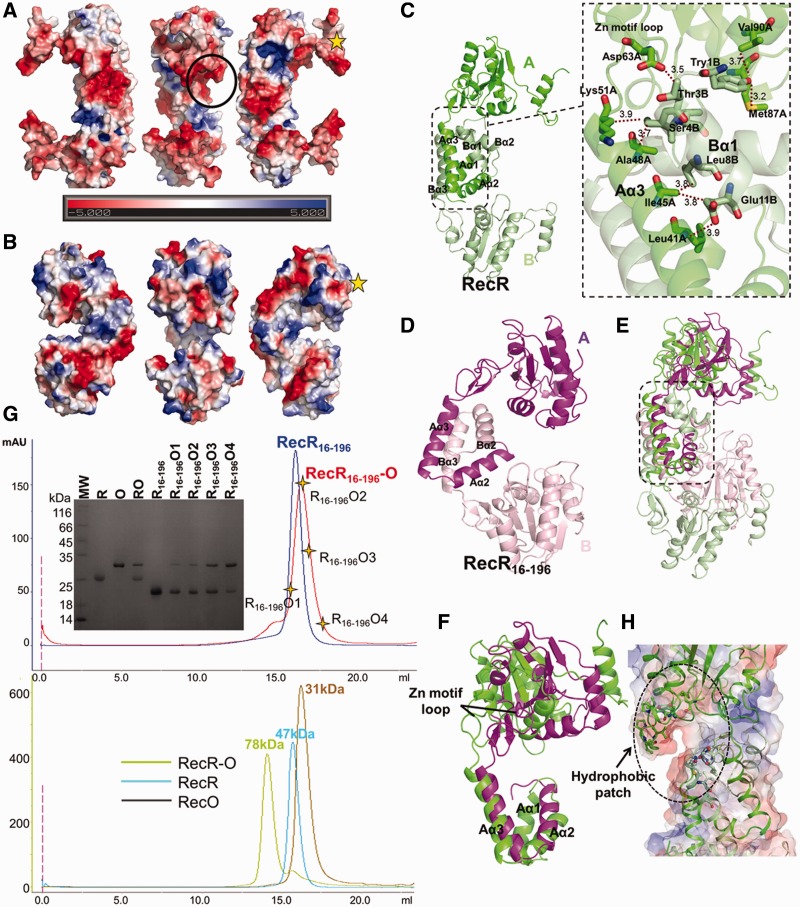
Structure of TTERecR_16-196_. Molecular surface of full-length TTERecR (**A**) and TTERecR_16-196 _(**B**) viewed in the same orientations. Electrostatic potential is indicated by different colours: blue is basic; red is acidic. (**C**) The structure of TTERecR N-N dimer (monomer A is green, monomer B is palegreen) is shown as a cartoon diagram. Expanded view of the circle, helix α1 of monomer B (palegreen) interacts with helix α3 and the zinc finger motif of monomer A (green). Interacting residues are shown as sticks. (**D**) The structure of the TTERecR_16-196 _N-N dimer (monomer A is purple, monomer B is light pink). (**E**) Superimposed structures of TTERecR_16-196 _N-N dimer (purple) and TTERecR N-N dimer (green). (**F**) Superimposed structures of the TTERecR_16-196_ monomer (purple) and TTERecR monomer (green). (**G**) Size-exclusion chromatography analysis of the interaction between TTERecR_16-196 _and TTERecO; TTERecR and TTERecO. (**H**) Hydrophobic patch (marked with a circle in Figure 3A) is formed by the hydrophobic loop06-121 and HhH domain of full-length TTERecR. The hydrophobic residues are shown as sticks.

Interestingly, TTERecR_16-196_ formed a dimer in solution as full-length TTERecR, but did not form a detectable complex with TTERecO in size-exclusion chromatography or SPR (*K*_D_ = 24.6 µM; Figure 2D; Figure 3G). We resolved the crystal structure of TTERecR_16-196_ at 2.8 Å resolution using molecular replacement. The space group of TTERecR_16-196 _was P21212, and the packing of the molecules in the unit cell was significantly different to full-length TTERecR. Two TTERecR_16-196 _monomers exist in a back-to-back conformation in the asymmetric unit, and two monomers from different asymmetric units form the TTERecR_16-196 _dimer by their N-terminal interaction, with an interface area of about 5581 Å^2^ (Supplementary Figure S3B).

The electrostatic surface potential of the TTERecR_16-196_ dimer and the TTERecR dimer was totally different. The most notable difference was that the interface area of the TTERecR N-N dimer had a negative potential, whereas the interface area of the TTERecR_16-196 _dimer had a positive potential (Figure 3A and B). Additionally, the C-terminus of TTERecR_16-196 _interacted with the Toprim domain to form a globular C-terminal region, which is apparent different from the C-terminus of TTERecR (Figure 3A and B). The TTERecR structure revealed that some residues of helix α1 (Ser4, Leu8, Leu12) in the HhH motif of one subunit had a hydrophobic interaction with some residues of helix α3 (Leu41, Ile45, Ala48, Lys51) in the HhH motif of the other TTERecR subunit (Figure 3C). Moreover, Thr3 interacted with Asp63 in the loop of the Zinc finger motif to stabilize the loop (Figure 3C). Superimposing the structures of TTERecR and TTERecR_16-196_, we found that helix α2 of TTERecR_16-196 _was 16° deviated to the helix α2 of TTERecR relatively, and helix α3 of TTERecR_16-196 _correspondingly moved 3 Å, and the zinc finger motif moved 5 Å towards the HhH motif (Figure 3F).

These structures indicated that the hydrophobic interactions between helix α1 of one monomer and helix α3 of the other monomer play a key role in maintaining the structure of the TTERecR N-N dimer (Figure 3C–E). In the full-length TTERecR N-N dimer, the hydrophobic residues Val106, Phe109 and Ile110 of the loop_106-121_ with the HhH domain created a hydrophobic patch for the interaction with TTERecO; however, this hydrophobic patch was destroyed in the TTERecR_16-196 _mutant, preventing the binding with TTERecO (Figure 3A and H). The helix α1 is essential for formation of the TTERecR N-N dimer and the TTERecOR complex, although helix α1 is located far away from the other three functional domains.

### A conserved basic Lys21 residue plays an important role in the RecR–RecO interaction

We investigated the ability of the TTERecR conserved residue mutants to interact with DNA or TTERecO. In contrast to reports, which have indicated that RecR_K21G_ has reduced or no ability to bind DNA (21,23,29), the TTERecR_K21G_ mutant had the same affinity to interact with plasmid DNA as full-length TTERecR (Supplementary Figure S4), and SPR demonstrated that the conserved residue mutant TTERecR_K21G_ and full-length TTERecR had an equally weak dsDNA-binding affinity (Supplementary Figure S5A and B). But TTERecR_K21G_ did not interact with TTERecO and could not form a TTERecO–TTERecR_K21G_ complex (Supplementary Figure S6). This result has not previously been reported.

**Figure 4.**
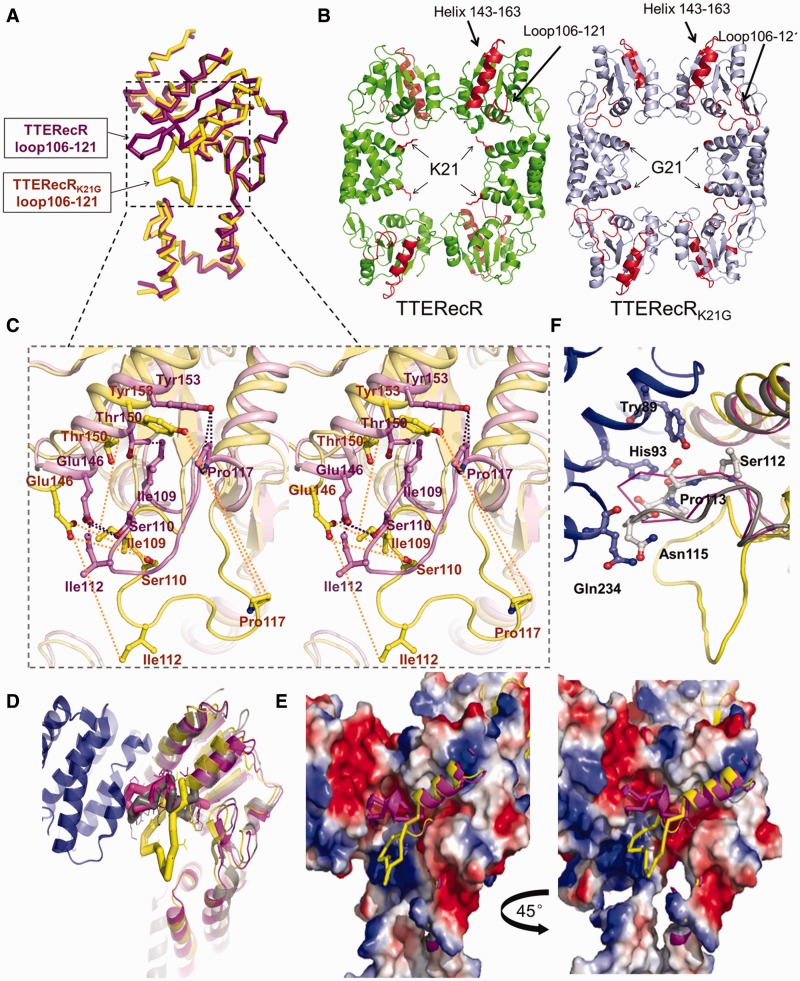
Structure of TTERecR_K21G_. (**A**) Superimposed TTERecR_K21G_ monomer (yellow) and TTERecR monomer (purple) structures as a ribbon diagram. (**B**) Comparison of the TTERecR_K21G_ tetramer structure (light blue) with TTERecR tetramer structure (green). Lys 21 of TTERecR and Gly21 of TTERecR_K21G_ are showed as sticks in red. Loop 106-121 and helix-α 143-163 is in red. (**C**) Superimposed TTERecR_K21G_ (yellow) and TTERecR (purple) structures, wall-eyed view showing the interactions between the helix-α (residues 143-163) and loop_106-121 _are broken in the TTERecR_K21G_ structure. Interacting residues are shown as sticks. (**D**) Superimposed structures of TTERecR_K21G_, TTERecR and drRecOR; drRecOR is coloured grey, other colours are as in A. (**E**) Electrostatic properties of the binding sites formed between TTERecR_K21G_ or TTERecR and drRecO. The complexes are shown as solvent-accessible surfaces coloured by electrostatic potential contoured at ±5 Kt/e (red, acidic; blue, basic). (**F**) Loop 106-121 of TTERecR (purple) and drRecR (grey) interact with the hydrophobic pocket of drRecO (blue). Interacting residues are shown as sticks.

The Lys21 residue of RecR is conserved in different species. It is hypothesized that the main function of Lys21 is to clamp DNA, and Lys21 has no influence on the binding to RecO (21). To investigate the significant difference observed in TTERecO binding after mutation of the TTERecR Lys21 residue to Gly, we solved the 2.8 Å crystal structure of the TTERecR_K21G_ mutant. The space group of TTERecR_K21G_ was F222, with only one monomer in the asymmetric unit (Figure 4A). Four crystallographic symmetric TTERecR_K21G_ mutants formed a ring-shaped tetramer similar to the full-length TTERecR structure (Figure 4B). The main difference between TTERecR and TTERecR_K21G _was the positional change of loop_106-121_. The offset angle of TTERecR_K21G_ reached 90° (Figure 4A and B), suggesting that loop_106-121 _plays an important role in the binding of TTERecR to TTERecO. Moreover, we found that mutation of Lys21 to Gly in TTERecR_K21G_ only lead to slight deviations in the polypeptide chains. However, this slight offset resulted in the helix-α (residues 143–163) moving 3-4 Å away from loop_106-121_, breaking the interactions between the helix-α (residues 143–163) and loop_106-121_. The distance between Tyr153 and Pro117 increased from 3.25 Å to 20.88 Å, and the distance between Glu146 and Ile112 increased from 3.45 Å to 13.01 Å; these shifts induced the loop_106-121 _to offset by 90° (Figure 4C). We superimposed the structural models of TTERecR, TTERecR_K21G_ and the drRecOR complex (PDB code: 2V1C), which revealed that loop_106-121 _was very similar in TTERecR and drRecR; but the loop_106-121 _of TTERecR_K21G_ completely deviated from the drRecR loop (Figure 4D). In the crystal structure of drRecOR complex, loop_106-121_ was like a wedge tightly bound to the hydrophobic pocket of drRecO, allowing RecR and RecO to form a stable complex (Figure 4D and E). In the crystal structure of TTERecR_K21G_, the loop (residues 117–122) flipped 90° away from RecO and lost their interaction with the link region (residues 78–88), the helix-α (residues 87–99) and the helix-α (residues 228–234), which are all in the hydrophobic pocket of RecO (Figure 4E and F). Based on this analysis, we speculated that the Lys21 is required for the interaction between TTERecR and TTERecO, and the loop_106-121 _in the Toprim domain is the most crucial site for the interaction with TTERecO.

### TTERecOR complex displays a clear preference for ssDNA

**Figure 5.**
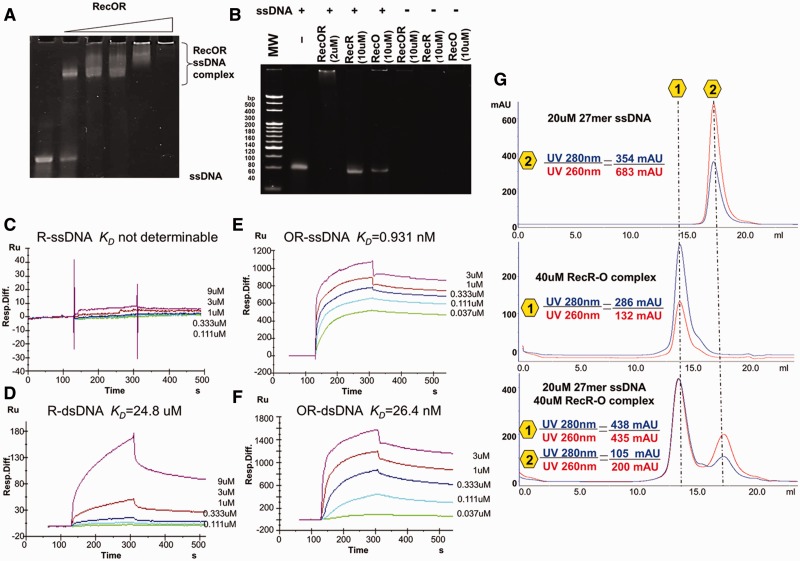
The TTERecOR complex displays a clear preference for ssDNA. (**A**) Analysis of the interaction between 60 mer ssDNA (2 µM) and different concentrations of TTERecOR (Lines 1–6: 0, 1, 2, 4, 10 and 50 µM, respectively) by native PAGE. (**B**) Analysis of the interaction between 60 mer ssDNA (2 µM) and TTERecR (10 µM), TTERecO (10 µM) or TTERecOR (2 µM) by native PAGE. (**C–F**) BIAcore biosensor analyses of TTERecR or TTERecOR binding to immobilized 60 mer ssDNA or 60 mer dsDNA at 25°C. The apparent *K*_D_ values were calculated from the kinetic *K*_D_ (M) = *K*_d_/*K*_a_. (**G**) Size-exclusion chromatography analysis of the interaction between 27 mer ssDNA and TTERecOR.

We used the EMSA assay to detect the interactions between TTERecR (10 µM), TTERecO (10 µM) and the TTERecOR (2 µM) complex with 60 mer ssDNA (2 µM), and found that only the TTERecOR complex had the capability to interact with ssDNA (Figure 5A and B). The DNA-binding affinities were determined by SPR. TTERecR did not interact with 60 mer ssDNA (Figure 5C), and only interacted weakly with 60 mer dsDNA (*K*_D_ = 24.8 µM; Figure 5D). TTERecO displayed high non-specific binding to the SA chip; therefore, the binding affinity of RecO for DNA could not be determined using this method. TTERecOR interacted strongly with 60 mer dsDNA (*K*_D_ = 26.4 nM) and ssDNA (*K*_D_ = 0.931 nM; Figure 5E and F). Thus, EMSA and SPR demonstrated that the TTERecOR complex stably bound to linear DNA, whereas the TTERecR and TTERecO did not stably bind linear DNA. Notably, TTERecOR had a much stronger binding affinity for ssDNA (*K*_D_ = 0.931 nM; Figure 5E) than dsDNA (*K*_D_ = 26.4 nM; Figure 5F), suggesting that the TTERecOR complex tends to bind ssDNA. We also obtained a stable sample of the TTERecOR complex with 27 mer ssDNA for further structural and functional research (Figure 5G). In addition, TTERecR_P16G_ and TTERecR_R25G_ had no binding affinity for 60 mer dsDNA. Surprisingly, TTERecR_P16G_ or TTERecR_R25G_ bound to TTERecO had an equally strong affinity for dsDNA or ssDNA as the full-length TTERecOR complex (Supplementary Figure S7). As a consequence, we speculated that the residues Pro16 and Arg25 of TTERecR are not part of the DNA-binding site, but maybe the recognition sites of DNA damage instead.

## DISCUSSION

It is notable that the HhH domain plays an important role in the interaction of RecR with RecO; however, it has been reported that only the HhH domain is responsible for DNA binding (21,25,26). Using structural and biochemical analysis of the TTERecR_16-196 _deletion mutant and TTERecR_K21G_ mutant, we demonstrated that the interaction between the entire HhH domains within the N-terminal regions of two TTERecR monomers is necessary for formation of a functional N-N RecR dimer. Slight movement of any helix in the HhH domain will firstly alter the electrostatic surface potential of TTERecR; secondly, lead to an enormous conformational change in the key loop_106-121_ (which is considerably far away from the TTERecR_K21G_ mutation site and forms the site of interaction between TTERecR and TTERecO); and finally, destroy the hydrophobic patch in the Toprim domain of TTERecR, which interacts with RecO. Our research also shows that the TTERecR dimer forms via an N-N terminal interaction, providing the basic functional unit for the interaction with TTERecO. Two N-N dimers of RecR form a ring-shaped tetramer via an interaction between their C-terminal regions. The ring-shaped TTERecR tetramer is preferred during crystal packing and is predicted to stabilize the RecR structure. The TTERecR_K21G_ crystal formed a similar ring-shaped tetramer to full-length TTERecR, but lost the ability to bind TTERecO. Therefore, we speculate that besides the interaction between 4 RecR:2 RecO in RecOR complex and 4 RecR:2 RecF in RecFR complex of the RecFOR repair pathway (29,30), it exists another mode of interaction, which is that the RecR N-N dimer interacts with RecO monomer.

**Figure 6.**
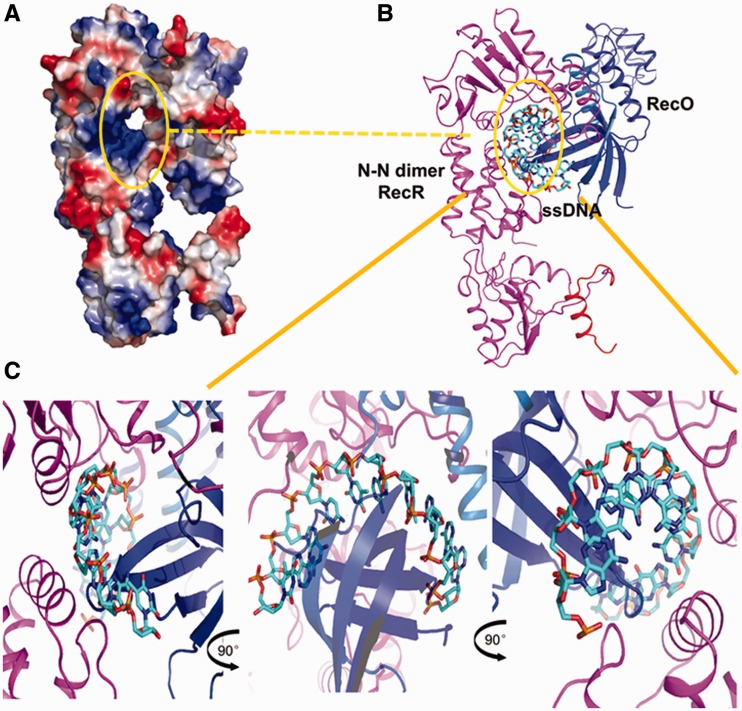
Model of ssDNA binding to TTERecOR. (**A**) Electrostatic properties of TTERecOR. The complex is shown as a solvent-accessible surface coloured by electrostatic potential contoured at ±5 Kt/e (red, acidic; blue, basic). (**B**) Hydrophobic channel (marked with a circle) formed between 1-23 aa of RecO and the N-terminal interaction sites of the TTERecR dimer. (**C**) ssDNA bound to the basic and hydrophobic channel of TTERecOR.

We also observed that TTERecR did not bind linear ssDNA and very weakly bound linear dsDNA; but when TTERecR interacted with TTERecO, the TTERecOR complex could bind linear DNA. The TTERecOR complex had a much stronger affinity for ssDNA. Reported models of the interactions of RecR or the RecOR complex with dsDNA show that dsDNA walks through the centre of the RecR ring (21,23,29,30). In our analysis, when the ring-shaped tetramer of RecR was bound to two RecO monomers, it was difficult to find a channel, which interacts with dsDNA in the centre of RecR ring and also interacts with the hydrophobic residues in the HhH domain (Supplementary Figure S8). We have constructed a structure model of the RecR N-N dimer and RecO monomer complex (Figure 6A) and RecOR and ssDNA complex (Figure 6B) by superimposing structures of the drRecOR complex and the TTERecR N-N dimmer based on our structural and functional analysis of TTERecR. This model shows that the N-terminus of RecO (α18-22 aa) interacts with the N-terminus of the RecR N-N dimer (1-30 aa), and this complex forms a channel within which a large number of positive charges and hydrophobic residues are distributed (Figure 6A). The diameter of this channel is about 15 Å, which is the same as the diameter of ssDNA. Therefore, we speculate that damaged ssDNA could pass through this channel (Figure 6A and B). Based on our structural and biochemical analysis, we constructed structure models of the RecOR:ssDNA complex (Figure 6B and C).

## ACCESSION NUMBERS

The structure factors and coordinates of TTERecR, TTERecR_16-196_ and TTERecR_K21G_ have been deposited into PDB under the accession codes: 3VDP, 3VE5 and 3VDU, respectively.

## SUPPLEMENTARY DATA

Supplementary Data are available at NAR Online: Supplementary Table 1 and Supplementary Figures 1–8.

## FUNDING

Major State Basic Research Development Program of China (973 Program) [No. 2011CB910302, 2011CB966303 and No. 2011CB911101]; National Natural Science Foundation of China [No. 31070684]. Funding for open access charge: Institute of Biophysics, Chinese Academy of Sciences.

*Conflict of interest statement*. None declared.

## Supplementary Material

Supplementary Data
