# Supplementary material for: RecOR complex including RecR N-N dimer and RecO monomer displays a high affinity for ssDNA
Source: Nucleic Acids Res. 2012 Sep 27;40(21):11115–25. doi: 10.1093/nar/gks889 (PMC3510498; doi:10.1093/nar/gks889)
Supplement: Supplementary Data [file supp_40_21_11115__index.html]

RecOR complex including RecR N-N dimer and RecO monomer displays a high affinity for ssDNA — RecOR complex including RecR N-N dimer and RecO monomer displays a high affinity for ssDNA — Supplementary Data 

# RecOR complex including RecR N-N dimer and RecO monomer displays a high affinity for ssDNA

## Supplementary Data

files

**Files in this Data Supplement:**

- Supplementary Data - pdf file
