# Supplementary material for: RecOR complex including RecR N-N dimer and RecO monomer displays a high affinity for ssDNA
Source: Nucleic Acids Res. 2012 Sep 27;40(21):11115–25. doi: 10.1093/nar/gks889 (PMC3510498; doi:10.1093/nar/gks889)
Supplement: Supplementary Data [file supp_gks889_nar-01564-m-2012-File008.pdf]

RecOR complex including RecR N-N dimer and RecO monomer displays a high affinity for ssDNA

SUPPLEMENTAL MATERIAL

This file includes:
Supplemental Table 1
Supplemental Figures 1 to 8

Supplemental Table 1.Statistics of crystallographic data collection

	RecR$_{1-166}$	RecR$_{1-180}$
A. *Data collection statistics*		
Space group	C21	P6222
Unit cell parameters		
a, *b*,*c* (Å)	841.87,90.05,83.13	294.81 295.33 100.27
α, β, γ (°)	β=92.15	
Resolution (Å)	5.2	3.0

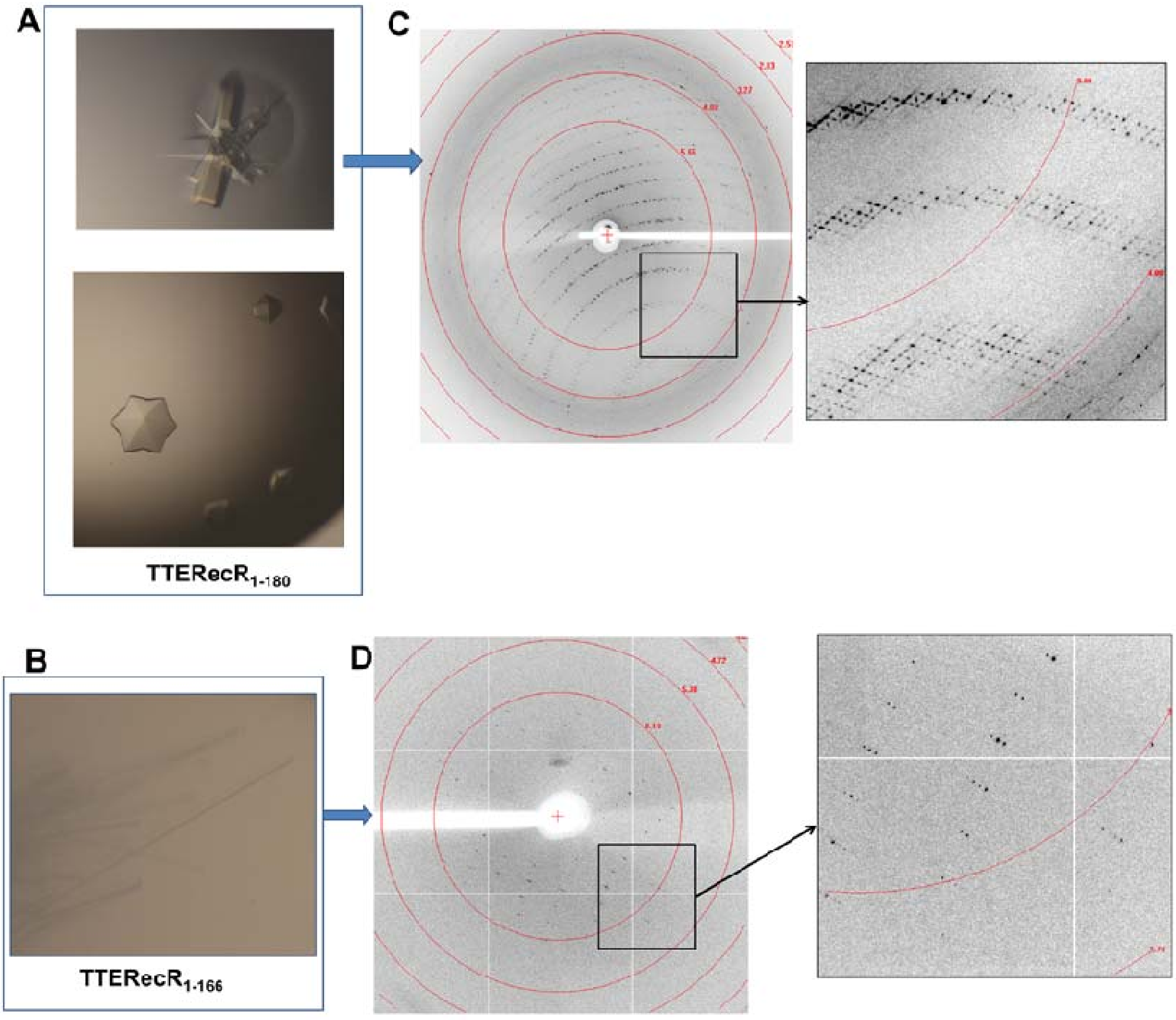

Supplemental Figure 1. Crystal and diffraction figure of TTERecR$_{1-166}$ and TTERecR$_{1-180}$ delete mutants. The crystals of TTERecR$_{1-180}$ delete mutant (A) and the crystal of TTERecR$_{1-166}$ delete mutant (B). The diffraction figure of TTERecR$_{1-180}$ delete mutant (C) and the diffraction figure of TTERecR$_{1-166}$ delete mutant (D).

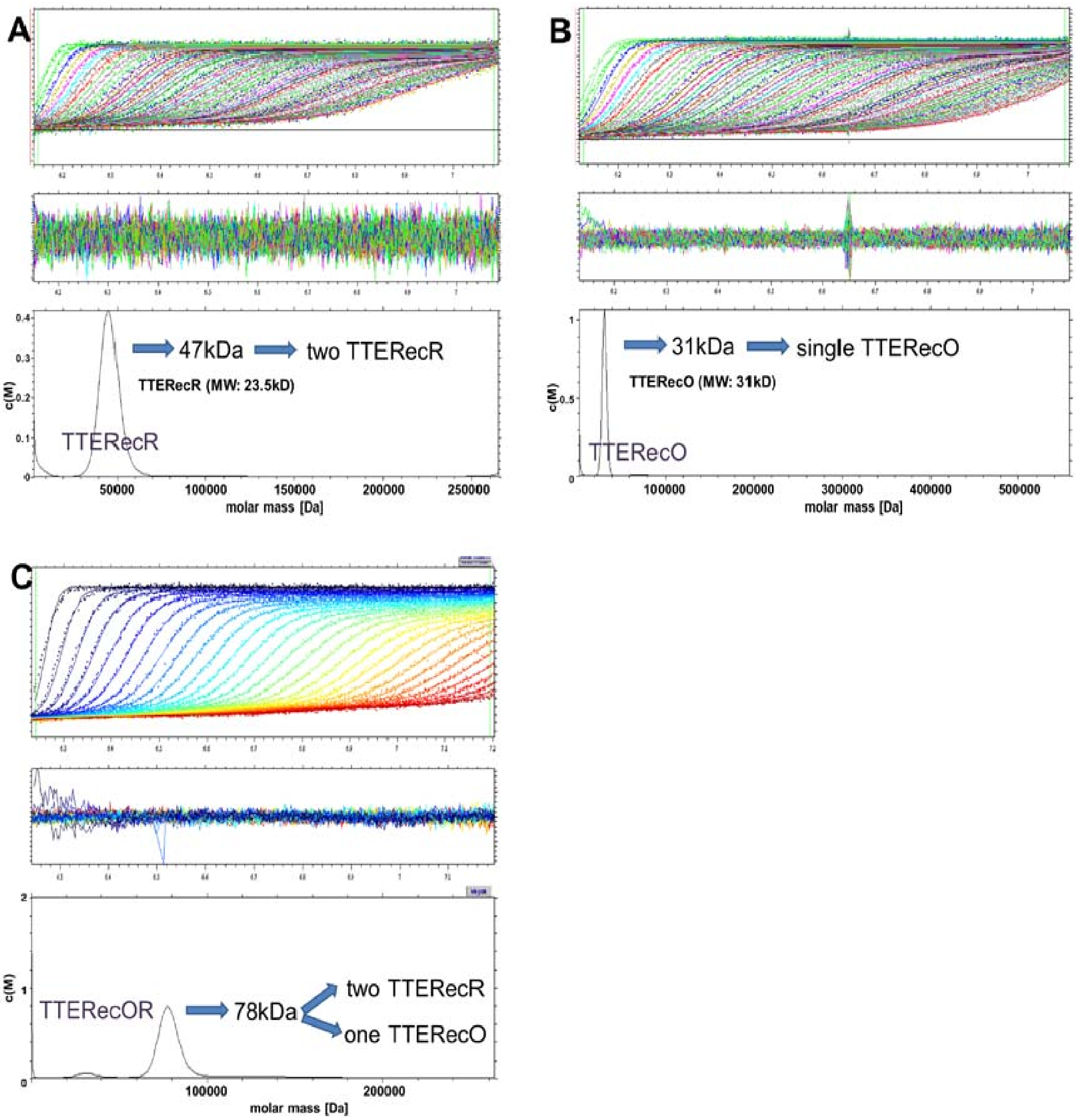

Supplemental Figure 2. Analytical ultracentrifugation analysis. The molecular weight of (A) TTERecR and (B) TTERecO were determined to be 47kDa and 31kDa separately indicating that TTERecR consists of two subunits (MW: 23.5kDa) in solution and TTERecO consists of one subunit (MW: 31kDa) in solution. (C) The molecular weight of TTERecOR complex was determined to be 78kDa indicating that the protein complex consists of two TTERecR molecules and one TTERecO molecule.

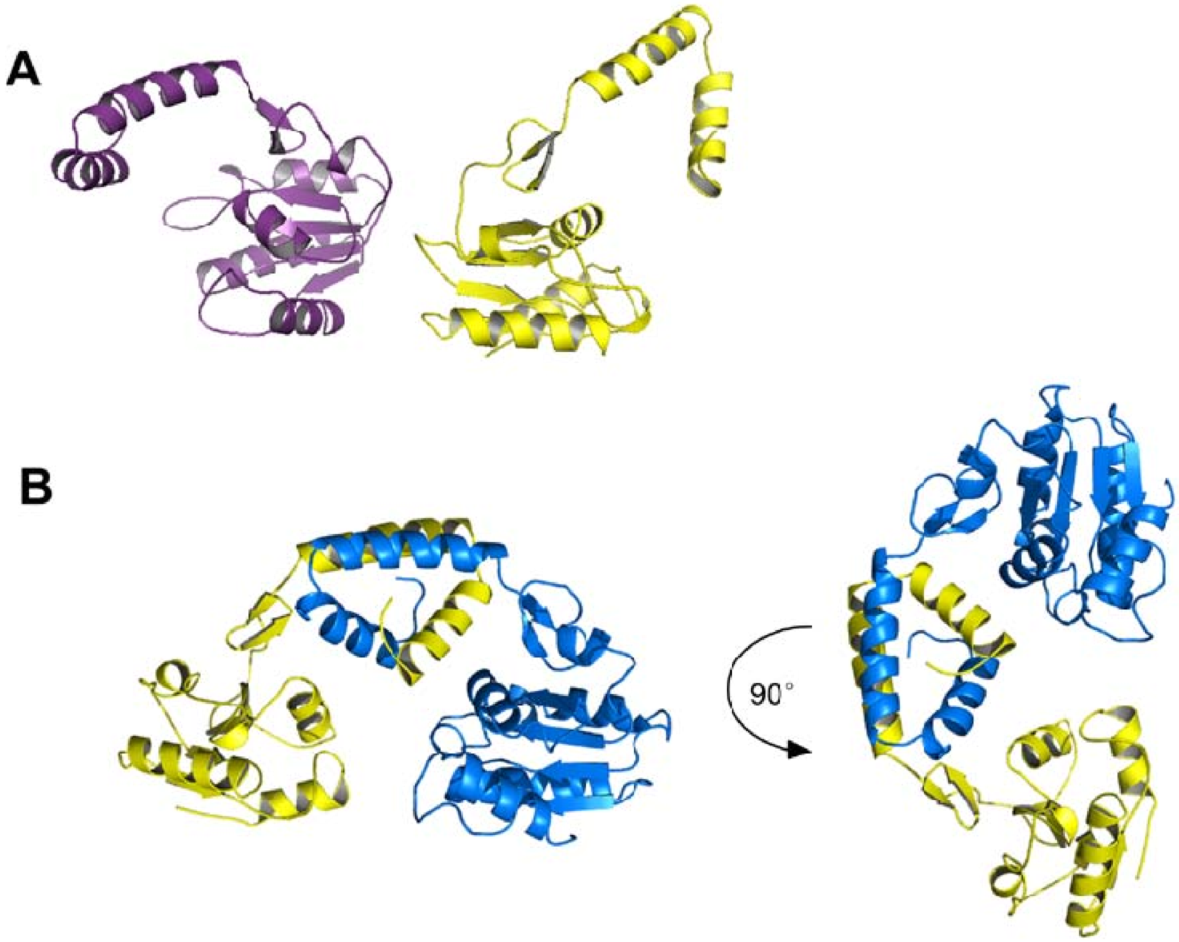

Supplemental Figure 3. Structure of TTERec$_{16\text{-}196}$ delete mutant. Asymmetric unit contains two TTERec$_{16\text{-}196}$ delete mutants (A). TTERec$_{16\text{-}196}$ N-N dimer is formed by two molecules which are symmetrical by crystallographic two-fold axis (B).

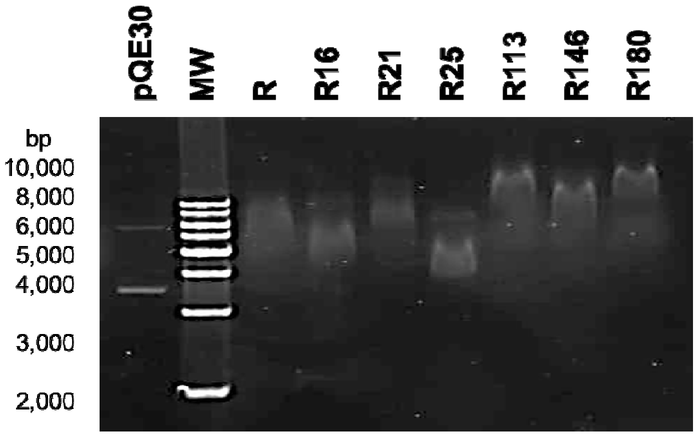

Supplemental Figure 4. Analysis of the interaction between the plasmid DNA (2uM) and TTERecR or the mutants (50uM) by native PAGE.

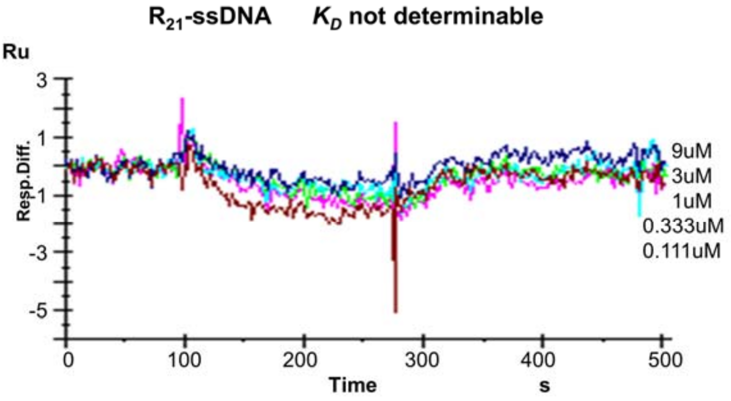

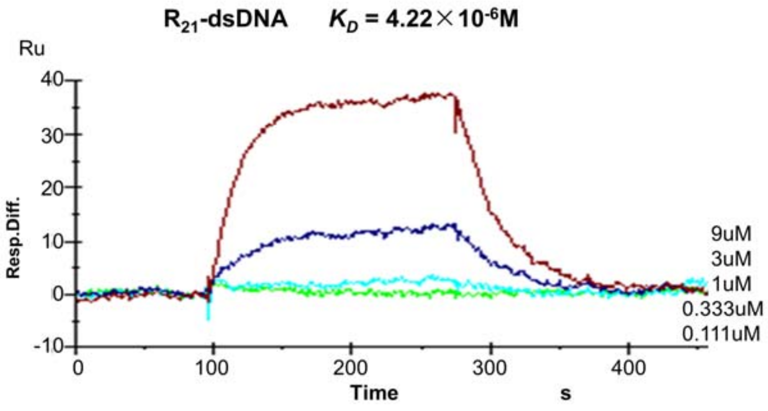

Supplemental Figure 5. BIAcore biosensor analyses of TTERecR$_{K21G}$ binding to 60 mers ssDNA or dsDNA at 25°C. The sensorgrams for 0.111uM, 0.333uM, 1uM, 3uM, 9uM concentration of TTERecR$_{K21G}$ mutant injected over surface coupled with 60merssDNA or dsDNA are shown. The apparent K_D was calculated from the kinetic K_D (M) $=K_d$ / K_a.

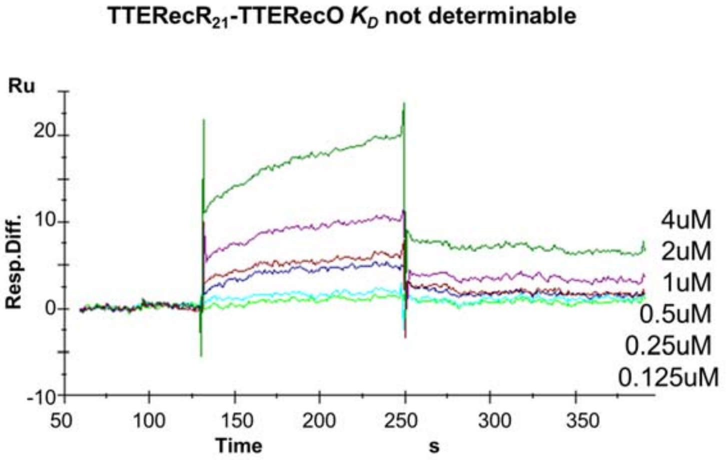

Supplemental Figure 6. BIAcore biosensor analyses of TTERecR$_{K21G}$ binding to TTERecO at 25°C. The apparent K_D was calculated from the kinetic K_D (M) =K_d / K_a. Compare with the binding ability of TTERecR to TTERecO, the binding signal is weak.

.

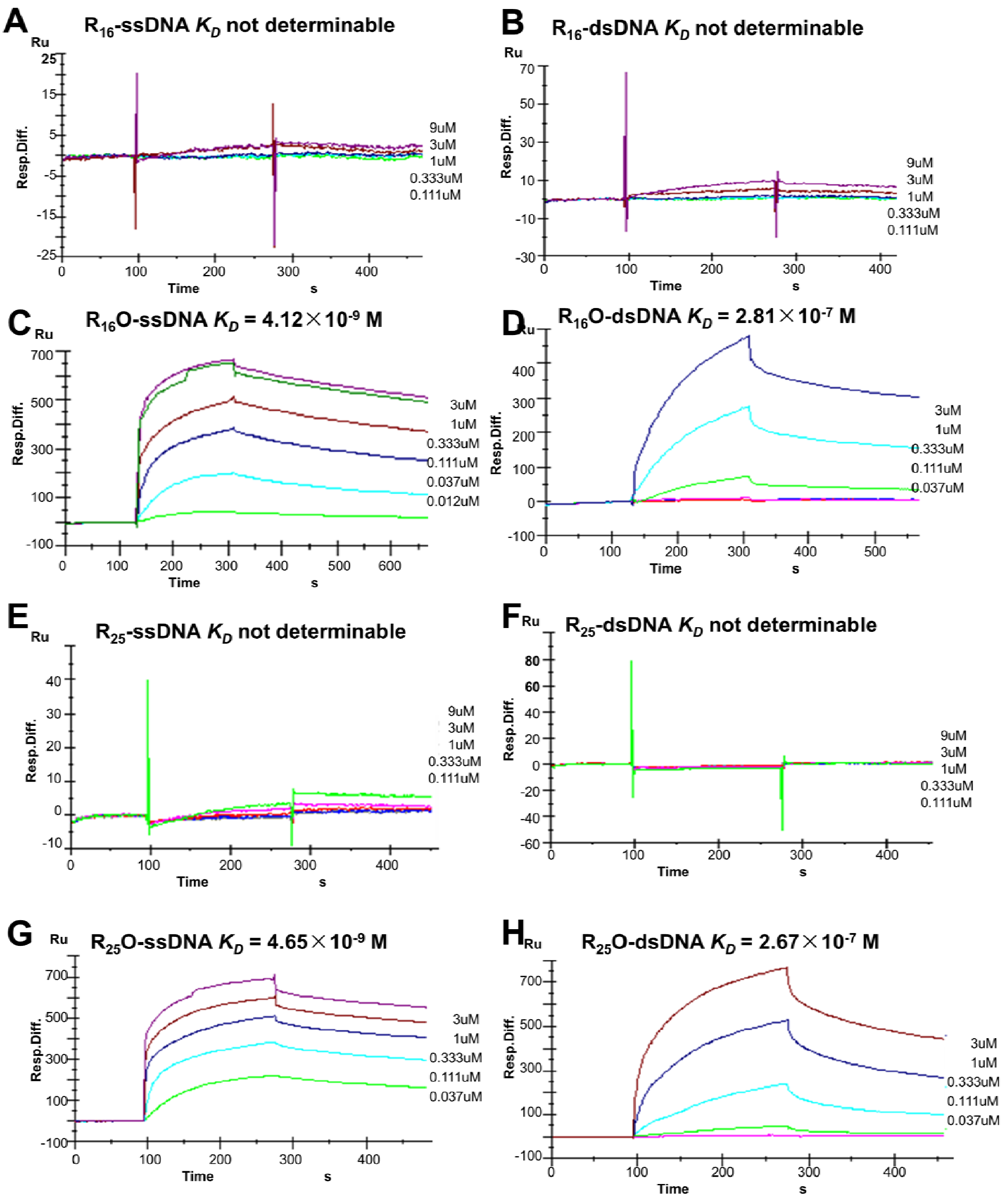

Supplemental Figure 7. BIAcore biosensor analyses of $TTERecR_{P16G}$, $TTERecR_{P16G}$-O complex, $TTERecR_{R25G}$ and $TTERecR_{R25G}$-O complex binding to 60 mers ssDNA or dsDNA. The sensorgrams for 0.111uM, 0.333uM, 1uM, 3uM, 9uM concentration of $TTERecR_{P16G}$ mutant (A, B) and $TTERecR_{R25G}$ mutant (E, F) injected over surface coupled with 60 mers ssDNA or dsDNA are shown, and the sensorgrams for 0.037uM, 0.111uM, 0.333uM, 1uM, 3uM of $TTERecR_{P16G}$-O complex (C, D) and $TTERecR_{R25G}$-O complex (G, H) injected over surface coupled with 60 mers ssDNA or dsDNA are shown at 25°C. For the apparent K_D was calculated from the kinetic K_D (M) $=K_d / K_a$.

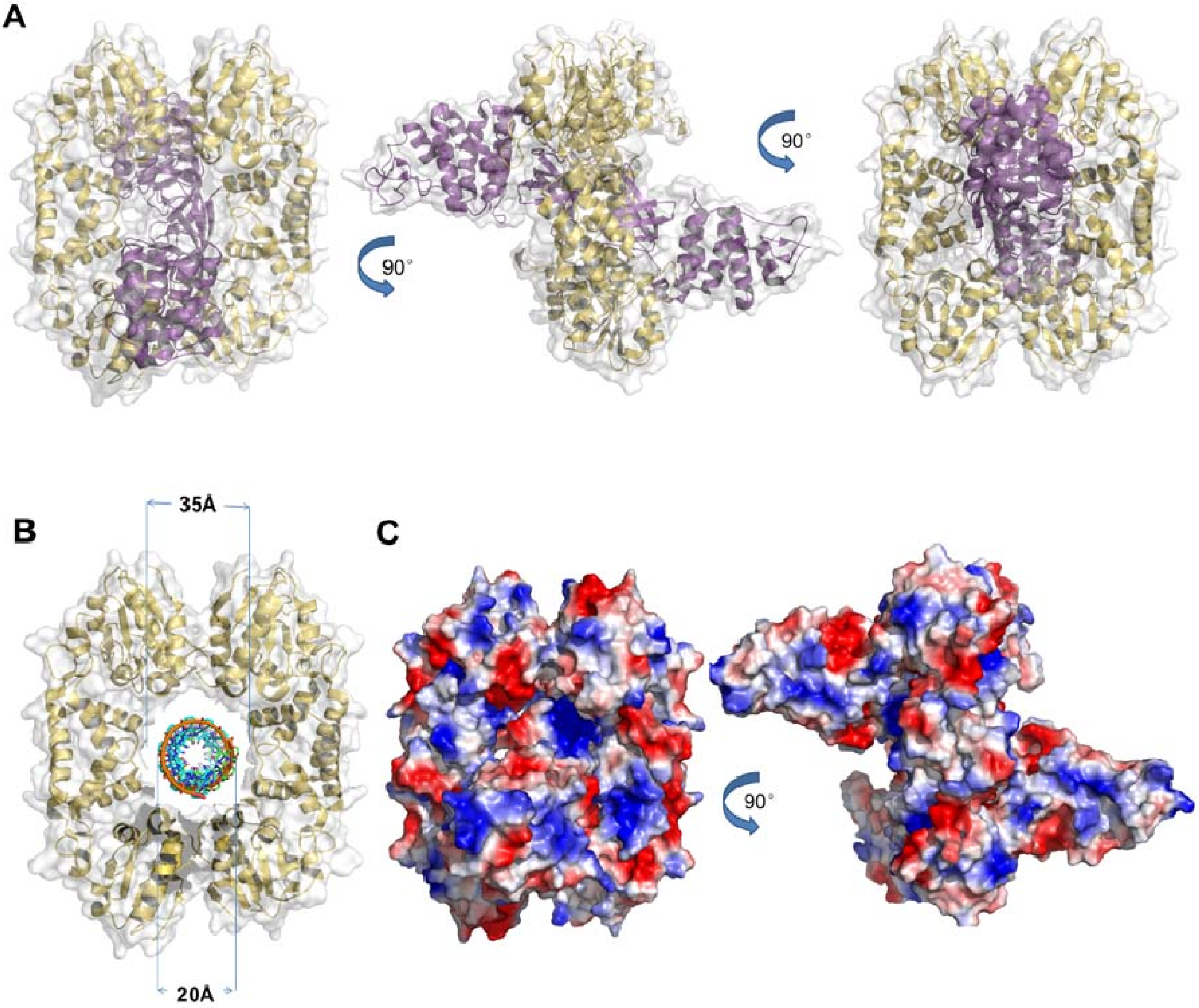

Supplemental Figure 8. Crystal structure of drRecOR complex (PDB code: 2V1C). (A)Ribbon representation of the heterohexameric drRecOR complex, as reconstituted by symmetry (drRecO is purple and drRecO is yellow). (B) Top view the model of drRecR with dsDNA, and dsDNA (green) is in the hole of drRecR. The diameter of the hole is 35 Å and the diameter of dsDNA is 20Å. (C) Illustration of the electrostatic surface potential of the heterotrimeric drRecOR assembly.
